# The mitochondrial NADH shuttle system is a targetable vulnerability for Group 3 medulloblastoma in a hypoxic microenvironment

**DOI:** 10.1038/s41419-023-06275-0

**Published:** 2023-11-30

**Authors:** J. Contenti, Y. Guo, A. Mazzu, M. Irondelle, M. Rouleau, C. Lago, G. Leva, L. Tiberi, I. Ben-Sahra, F. Bost, N. M. Mazure

**Affiliations:** 1 Université Côte d’Azur, INSERM U1065, C3M, 151 Route de St Antoine de Ginestière, BP2 3194, CEDEX 03, 06204 Nice, France; 2 Pasteur II Hospital, Department of Emergency Medicine, University Hospital Center, 30 voie Romaine, 06000 Nice, France; 3 Université Côte d’Azur, Laboratoire de PhysioMédecine Moléculaire - LP2M, CNRS-UMR 7370, Faculty of Medicine, 28 ave de Valombrose, 06107 Nice Cedex 02, France; 4 Armenise-Harvard Laboratory of Brain Disorders and Cancer, Department of Cellular, Computational and Integrative Biollogy - CIBIO, University of Trento, Via Sommarive 9, 38123 Trento, Italy; 5 Northwestern University Feinberg School of Medicine, Robert H. Lurie Cancer Center, 303 East Superior Street, Chicago, IL 60611 USA

**Keywords:** Paediatric cancer, Cancer metabolism, Cancer microenvironment, Target identification, Apoptosis

## Abstract

Medulloblastoma is a cancerous brain tumor that affects mostly children. Among the four groups defined by molecular characteristics, Group 3, the least well characterized, is also the least favorable, with a survival rate of 50%. Current treatments, based on surgery, radiotherapy, and chemotherapy, are not adequate and the lack of understanding of the different molecular features of Group 3 tumor cells makes the development of effective therapies challenging. In this study, the problem of medulloblastoma is approached from a metabolic standpoint in a low oxygen microenvironment. We establish that Group 3 cells use both the mitochondrial glycerol-3 phosphate (G3PS) and malate-aspartate shuttles (MAS) to produce NADH. Small molecules that target G3PS and MAS show a greater ability to decrease cell proliferation and induce apoptosis specifically of Group 3 cells. In addition, as Group 3 cells show improved respiration in hypoxia, the use of Phenformin, a mitochondrial complex 1 inhibitor, alone or in combination, induced significant cell death. Furthermore, inhibition of the cytosolic NAD+ recycling enzyme lactate dehydrogenase A (LDHA), enhanced the effects of the NADH shuttle inhibitors. In a 3D model using Group 3 human cerebellar organoids, tumor cells also underwent apoptosis upon treatment with NADH shuttle inhibitors. Our study demonstrates metabolic heterogeneity depending on oxygen concentrations and provides potential therapeutic solutions for patients in Group 3 whose tumors are the most aggressive.

## Introduction

Pediatric cancers are a leading cause of death by disease in children and young people [1]. Medulloblastoma (MB), a cancerous brain tumor in the cerebellum, is the most common malignant brain tumor in children, accounting for 20% of all pediatric central nervous system tumors. Medulloblastoma is a fast-growing cancer that often spreads to other parts of the brain and spinal cord. MBs are divided into four subgroups based on the molecular characteristics of the tumor cells: wingless (WNT), sonic hedgehog (SHH), Group 3, and Group 4 [2–4]. While subgroup WNT has a good prognosis, and SHH and Group 4 tumors have intermediate prognoses, clinical outcomes for Group 3 MBs are less favorable [5]. A better understanding of the molecular basis of medulloblastoma has led to improved survival rates in recent years. However, treatment options are still lacking, especially for Group 3 patients. A major obstacle to developing more effective therapeutic strategies has been the management of MBs as a uniform disease. Therefore, we consider the molecular and clinical characteristics of MBs to specifically address the treatment of Group 3 tumors.

Hypoxia plays an important role in a broad range of solid tumors [6, 7]. Cancer cells undergoing hypoxia reprogram their metabolism to trigger survival mechanisms and promote tumor progression [8]. One of these adaptations occurs by stimulating central carbon metabolism through the Warburg effect [9, 10]. Central carbon metabolism comprises glycolysis, the pentose phosphate pathway, the tricarboxylic acid cycle (TCA), and fatty acid metabolism. The shift from oxidative catabolism (energy release) to anabolic metabolism (biomass synthesis) is controlled by critical cofactors that enable the disposal of excess electrons. These cofactors include redox couples, such as nicotinamide adenine dinucleotide (NAD^+^/NADH) and nicotinamide adenine dinucleotide phosphate (NADP^+^/NADPH), which are essential for maintaining cellular redox homeostasis and modulating several biological processes, including cellular metabolism [11–15].

Cells employ three different mechanisms to oxidize NADH and regenerate cytosolic NAD^+^ [16]. The first two mechanisms rely on malate dehydrogenase 1 (MDH1), glutamic-oxaloacetate transaminase 1/2 (GOT1/2), and cytosolic and mitochondrial glycerol 3-phosphate dehydrogenase (cGPDH and mGDPH), which are components of the malate-aspartate shuttle (MAS) and glycerol 3-phosphate shuttle (G3PS), respectively. The third mechanism to oxidize cytosolic NADH is to reduce pyruvate to lactate with lactate dehydrogenase (LDHA/B). Under normoxic conditions, the MAS and G3PS are more efficient ways to regenerate NAD^+^ than LDHA/B because they enable glycolysis-derived pyruvate to be oxidized in mitochondria. The reducing equivalents transferred from cytosolic NADH to mitochondrial NAD^+^ by the MAS and G3PS are ultimately used to reduce oxygen at complex IV of the electron transport chain. Thus, when oxygen is limited, the only mechanism for regenerating cytosolic NAD^+^ is through the LDH reaction that produces lactate.

**Fig. 1:**
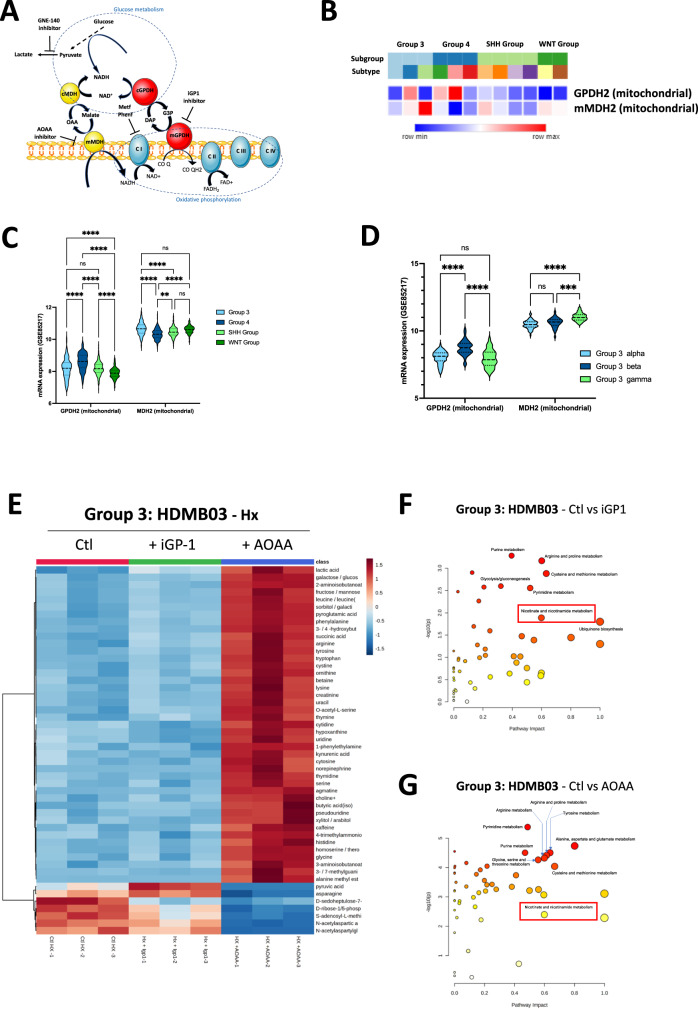
Mitochondrial G3P and MAS shuttles are used differently by Group 3 under hypoxia. **A** Schematic representation of the metabolic pathways driven by G3PS and MAS shuttles. Specific inhibitors are represented: iGP1 (mGPDH), AOAA (MDH2), and GNE-140 (LDHA). **B** Heatmap of the RNA expression of *GPDH2* and *MDH2* genes from GSE85217 comprising 763 primary samples from MB patients from Group 3, Group 4, SHH Group, and WNT Group MB [19]. Expression of the genes was compared using Phantasus (v1.19.3). **C** Violin plot of the RNA expression of *GPDH2* and *MDH2* genes from GSE85217 comprising 763 primary samples from MB patients from Group 3, Group 4, SHH Group and WNT Group MB [19]. Expression of the genes was compared using Prism 9 version 9.5.0. **D** Violin plot of the RNA expression of *GPDH2* and *MDH2* genes from GSE85217 comprising 763 primary samples from MB patients from Group 3 alpha, Group 3 beta and Group 3 gamma MB [19]. Expression of the genes was compared using Prism 9 version 9.5.0. **C**, **D** The 2-way ANOVA is used to determine statistically difference between the different groups. Not significant (ns), **p = 0.0014, ***p = 0.001 and ****p < 0.0001. **E** Steady-state metabolite profile of HDMB03 cells subjected to hypoxia for 72 h in the absence (Ctl - Hx), or presence of i-GP1 or AOAA. Intracellular metabolites from four independent samples per condition were profiled by LC/MS-MS, and those significantly altered in treated cells, relative to control cells, are shown as row-normalized heatmaps ranked according to log_2_ fold-change (treated/untreated). Pathway analysis of HDMB03 cells subjected to hypoxia in the absence (Ctl - Hx), or presence of i-GP1 (**F**) or AOAA (**G**).

To study the role of MAS and G3PS in Group3 cells in terms of cancer cell fitness, we tested the effect of mitochondrial GPDH inhibitor (iGP-1) targeting G3PS as well as aminooxyacetate acid (AOAA) targeting GOT1/2 (Fig. 1A). We tested the actions and inhibitions of these metabolic mechanisms in Group 3 cells (HDMB-03 and D-458) in different oxygen microenvironments. We consider cerebellum microenvironments of 6% oxygen concentration (physiological control conditions, physioxia, Phx) [17, 18] and 1% oxygen concentration (physiopathological conditions, hypoxia, Hx), as well as 21% (normal laboratory atmospheric conditions, normoxia, Nx). Under hypoxic conditions, we also tested the effect of an LDHA/B inhibitor (GNE-140) on Group 3. We show that co-targeting MAS and G3PS induces substantial antiproliferative effects on Group 3 cells. Mechanistically, we show that the combination induced apoptosis of Group 3 tumors. Moreover, the addition of the LDHA inhibitor potentiated the effects of the different NADH shuttle inhibitors. Overall, we demonstrate that the NADH-generating pathways maintain the Group 3 MB tumor growth and progression and can therefore be targeted to fight these MBs with poor prognosis.

## Materials and methods

### Cell culture

The HDMB-03 cell line was purchased from the DSMZ (ACC740). The D-458 cell line was provided by Dr. C. Pouponnot (Institut Curie - France). Both cells were grown in Dulbecco’s Modified Eagle’s Medium (DMEM) (Gibco-BRL) supplemented with 20% fetal bovine serum with penicillin G (50U/ml) and streptomycin sulfate (50 µg/ml).

An INVIVO_2_ 200 anaerobic workstation (Ruskinn Technology Biotrace International Plc) set at 6% and 1% oxygen, 94% nitrogen and 5% carbon dioxide were used for hypoxic conditions.

### Pharmacological inhibitors and chemicals

Phenformin, rotenone, antimycin A, oligomycin, and 2, 4-Dinitrophenol (DNP) were from Sigma (St. Louis, USA), iGP-1 from Merck (Darmstadt, Germany) and AOAA from Selleckchem (Houston, USA) and GNE-140 from MedChemExpress (Monmouth Junction, USA).

### Data sources

Affimetrix Human Gene 1.1 ST Array profiling of 763 primary medulloblastoma samples (GSE85217) was used for identification of Medullobastoma subtypes [19].

### Colony-forming assay

Cells (5000–10,000) were plated on 60-mm dishes and incubated at 37 °C, 5% CO_2_ for colony formation. After 7 days, colonies were fixed with 10% (*v*/*v*) methanol for 15 min and stained with 5% Giemsa (Sigma, St. Louis, USA) for 30 min for colony visualization.

### Enzymatic assays

The Glycerol 3-Phosphate colorimetric Assay Kit (MAK207), NAD/NADH quantitation kit (MAK037), and Malate assay kit (MAK067) were used according to the manufacturer’s instructions (Sigma Aldrich - St. Louis, USA).

### Cell counting for viability and proliferation assessment

Cells were plated at 100,000 cells/well and treated the following day. At specific times, cells were detached using trypsin-EDTA, suspended in their conditioned medium and evaluated for viability and proliferation using an automatic cell counter (Advanced Detection Accurate Measurement system, Digital bio, NanoEnTek Inc., Seoul, Korea).

### Organoid maintenance, modification, and injection

Human iPS cells (iPSC, ATCC-DYS0100) were maintained in self-renewal on a layer of geltrex (Gibco, A14133-01), in E8 Basal Medium (Gibco, A15169-01) supplemented with E8 supplement (50 X). All cells were mycoplasma-free. iPSCs were dissociated with EDTA (Invitrogen) 0.5 mM, pH 8.0, for 3 min incubation, to maintain cell clusters. Cerebellar organoids were cultured as described by Muguruma et al. [20] and Ishida et al. [21] and were electroporated at 35 days of differentiation protocol with 16.6 μg pCAG PiggyBac (PBase), 16.6 μg of pPB CAG-YFP (Venus), 33.3 μg pPB CAG-MYC and 33.3 μg pPB CAG-Otx2resuspended in Buffer 5 [22]. Organoids were transferred inside the electroporation cuvettes (VWR, ECN 732-1136, 2 mm), and electroporation was performed with the Gene Pulser XcellTM.

Organoids were treated from days 71 or 79 of differentiation, with three doses of 100 μM iGP1, 1 mM AOAA, 5 µM GNE-140, 100 µM Phenf, and two combos iGP1/GNE-140/AOAO, iGP1/GNE-140/Phenf or DMSO as a control. After the drug treatment, organoids were fixed in PFA 4%, permeabilized with 0.5% Triton X-100 for 30 min and then blocked for 1 h at room temperature. Primary antibodies against cleaved Caspase 3 (Sigma, AB3623 - 1:400) were incubated overnight and a secondary antibody was coupled to AlexaFluor 594 for 1 h (Invitrogen, A11012 - 1:200). Annexin V (AAT Bioquest, 20074 – 1:200) was incubated 24 h before imaging. Tumoroids were imaged with a Nikon confocal spinning disk microscope. Images were processed with Imaris for 3D reconstruction.

### Respirometry and extracellular acidification

The cellular oxygen consumption rate (OCR) and extracellular acidification rate (ECAR) were obtained using a Seahorse XF96 extracellular flux analyzer from Seahorse Bioscience (North Billerica, MA, USA) following the manufacturer’s instructions. OCR and ECAR were measured in real time in Nx, Phx, or Hx. Cells were deprived of glucose for 1 h, then glucose (G–10 mM), oligomycin (O–1 µM), 2,4-Dinitrophenol (DNP–10 µM), and Rotenone + Antimycin A (R/A–1 µM) were injected at the indicated times. Protein standardization was performed after each experiment, with no noticeable differences in protein concentration and cell phenotype.

### Phenotype MicroArray on Omnilog™ analyzer

Metabolic profiling was studied by using the Omnilog^®^ Phenotype Microarray™ system (Biolog, Hayward, CA, USA) evaluating the cell’s ability to metabolize 367 substrates. Cells were cultured for 48 h in normoxia or hypoxia, then transferred at seeding densities of 20,000 cells/well to the PM-M1 to 4 plates in a phenol red-free RPMI-1640-based medium depleted of carbon energy sources (IFM1 medium, Biolog Inc., Hayward, CA, USA), supplemented with 0.3 mM glutamine, 5% FCS, 100 U/mL penicillin, and 100 µg/mL streptomycin. Cells were then incubated for 24 h at 37 °C under 5% CO_2_ in hypoxia or normoxia before adding Biolog Redox Dye Mix MA, sealing the plate with tape to prevent gas transfer, and incubating at 37 °C in the Omnilog^®^ automated incubator-reader (Biolog Inc., Hayward, CA, USA) for 24 h to kinetically measure tetrazolium reduction. Data was collected using PMM Kinetics software with subtraction of the average values of three negative control wells (background), then, analysis was conducted with the opm package in R (Version 3.4.4).

### Steady-state metabolomics

To determine the relative levels of intracellular metabolites, extracts were prepared and analyzed by LC/MS-MS. Triplicate 10-cm plates (∼80% confluent) were incubated in Hx for 72 h. Metabolites were extracted on dry ice with 4 mL 80% methanol ( −80 °C), as described previously [23]. Insoluble material was pelleted by centrifugation at 3000 *g* for 5 min, followed by two subsequent extractions of the insoluble pellet with 0.5 mL 80% methanol, with centrifugation at 16,000 *g* for 5 min at 4 °C. The 5-mL metabolite extract from the pooled supernatants was dried down under nitrogen gas using an N-EVAP (Organomation Associates, Inc). Dried pellets were resuspended using 20 μL HPLC-grade water for mass spectrometry. A 7-μL sample was injected and analyzed using a 5500 QTRAP triple quadrupole mass spectrometer (AB/SCIEX) coupled to a Prominence UFLC HPLC System (Shimadzu) via selected reaction monitoring of a total of 300 endogenous water-soluble metabolites for steady-state analyses of samples [24]. The normalized areas were used as variables for the univariate statistical data analysis. All univariate analyses and modeling on the normalized data were carried out using Metaboanalyst 4.0 (http://www.metaboanalyst.ca). Univariate statistical differences in the metabolites between two groups were analyzed using a two-tailed Student *t*-test.

### Immunoblotting

Cells were lysed in 1.5x SDS buffer and the protein concentration determined using the BCA assay. 40 µg of protein from whole cell extracts was resolved by SDS-PAGE and transferred onto a PVDF membrane (Millipore). Membranes were blocked in 5% non-fat milk in TN buffer (50 mM Tris-HCl pH 7.4, 150 mM NaCl) and incubated in the presence of the primary and then secondary antibodies in 5% non-fat milk in TN buffer. Rabbit monoclonal anti-GPD2 antibody (ab188585) was from Abcam (Paris, France). Rabbit monoclonal anti-MDH2 antibody (D8Q5S) was from Cell Signaling technology. Mouse anti-b-tubulin, HSP90 and b -actin were from Sigma. ECL signals were normalized to either b-tubulin or HSP90. After washing in TN buffer containing 1% Triton-X100 and then in TN buffer, immunoreactive bands were visualized with the ECL system (Amersham Biosciences).

### Immunofluorescence

For live imaging, human MB tumor organoids were observed with an Evos optical microscope with GFP fluorescence (Venus). Immunofluorescence staining was performed in 96-well flat-bottom microplates (Greiner Bio-One: 655090). Blocking and antibodies solutions consisted of PBS supplemented with 2% Fetal Bovine Serum. Human MB tumor organoids were harvested and washed once with ice cold culture medium. Human MB tumor organoids were post-fixed for 1 h in 4% PFA, permeabilized with 0.5% Triton X-100 (SIGMA: T8787) for 30 min and then blocked for 1 h at room temperature (RT). Primary antibody against Caspase 3 active (cleaved) form (Sigma-Aldrich: AB3623, 1:400) was incubated overnight. Secondary antibody coupled to AlexaFluor 594 (Invitrogen: A11012, 1:200) was incubated for 1 h at RT. Human MB tumor organoids were mounted and imaged with a Nikon confocal microscope. Images were processed with Imaris for 3D reconstruction and measurement.

### Statistics

All values are the means ± SEM. Statistical analyses were performed using the ordinary oneway ANOVA and 2-way ANOVA tests in Prism. The *p* values are indicated. All categorical data used numbers and percentages. Quantitative data were presented using the median and range or mean. All statistical tests were two-sided, and *p* values < 0.05 indicated statistical significance while *p*-values between 0.05 and 0.10 indicated a statistical tendency.

## Results

### Differential utilization of the G3P and MA shuttles by Group3 under hypoxia

Three different mechanisms are mainly used to oxidize NADH in the cytosol, thereby regenerating NAD+ for GAPDH. The first two mechanisms rely on the cGPDH and MDH1, which are components of the glycerol 3-phosphate shuttle (G3PS) and malate-aspartate shuttle (MAS), respectively (Fig. 1A). We consulted the Affymetrix Human Gene 1.1 ST array profiling of 763 primary medulloblastoma samples (GSE85217) [19], which showed that *GPD2* gene expression, encoding mGPDH, and *MDH2*, encoding mitochondrial malate dehydrogenase (MDH2), were different between the four groups (Fig. 1B). *GPD2* expression in Group 3 MB was significantly lower than in Group 4 MB but significantly higher than in the WNT Group (Fig. 1C). Across all groups, *MDH2* expression was higher compared with *GPDH2* expression. Moreover, Group 3 MB expressed *MDH2* more strongly than Group 4 and SHH MB. As three distinct subtypes of Group 3 MB emerged from the analysis of Cavalli et al. [19], alpha, beta, and gamma, with Groups 3 alpha and 3 beta having a more favorable prognosis than Group 3 gamma, we also checked *GPD2* and *MDH2* gene expressions in these different subtypes. *GPDH2* expression was higher in Group 3 beta but *MDH2* was significantly higher in Group 3 gamma (Fig. 1D). Such heterogeneity of expression within the different groups and subtypes supports the value of studying the impact of inhibiting these two metabolic pathways. Moreover, these results may already suggest that MDH2 plays a more important role than GPDH2 in the aggressiveness of Group 3 MB tumor cells and especially in the gamma subtype.

We then decided to focus our attention on Group 3 MB, which is the most lethal of the four groups. HDMB03 and D-458 cells, representative of Group 3 MBwere first exposed for 72 h to Nx (21% O_2_), Phx (6%) and Hx (1%) microenvironments. We observed no significant increase in mGPDH and MDH2 mRNA expressions after 72 h in Group 3 MB cells (Supplementary Fig. S1A, B). However, mGPDH protein expression was decreased depending on oxygen concentration only in HDMB03 cells (Supplementary Fig. S1C). MDH2 protein expression was also decreased in HDMB03 cells but not in D-458 cells (Supplementary Fig. S1D).

We then evaluated the glycerol-phosphate and malate-aspartate pathways with the OmniLog Phenotype MicroArray to obtain the mitochondrial metabolic signature of HDMB03 and D-458 cells under Nx, Phx, and Hx using MitoS1 plates. HDMB03 and D-458 (Supplementary Fig. S2A) cell growth was a function of decreasing oxygen. HDMB03grew on malate under all three conditions tested (Nx, Phx, and Hx) whereas D-458 cells were only able to utilize malate under Phx and Hx conditions (Supplementary Fig. S2B). We also observed that other compounds associated with malate, such as acetyl L carnitine, keto-isocaproic, and leucine were readily used by HDMB03 under Hx.

To test the activity of these two shuttles and potential therapeutic purposes, we used known inhibitors (iGP1) and aminooxyacetate (AOAA) to block mGPDH and mMDH pathways, respectively. Similar G3P accumulation and NAD/NADH ratio were observed in the presence of both siRNA targeting mGPDH and iGP1 in HDMB03 Group 3 MB cells (Supplementary Fig. S2C, D). Malate concentration was significantly reduced in the presence of AOAA compared to siRNA targeting MDH2 (Supplementary Fig. S2E). These results supported our chemical approach. We then performed an unbiased metabolomic profiling on HDMB03 (Group 3) under Hx (Fig. 1E). HDMB03 were untreated or treated with specific inhibitors of mGPDH (iGP1) or mMDH (AOAA) that both impacted on nicotinate and nicotinamide metabolism (Fig. 1E–G). Of the 262 small metabolites identified by LC/MS-MS), the steady-state levels of 50 metabolites were significantly altered in both cells treated with iGP1 or AOAA relative to control cells (*P* < 0.02). The profiles obtained are clearly different. HDMB03 cells produced more pyruvate and asparagine, suggesting that they may promote glycolysis and the TCA cycle. iGP1 clearly impacted HDMB03 metabolism via a reduction in the production of D-sedoheptulose-7-phosphate, D-ribose-1/5-phosphate, and S-adenosyl-methionine, suggesting a direct impact on the lipopolysaccharide, pentose phosphate and s-adenosylmethionine metabolic (SAM) pathways (Fig. 1E and Supplementary Fig. S2F). In contrast, AOAA dramatically reversed the metabolism of the HDMB03 cells, which then seem to use or accumulate highly mitochondrial metabolites.

These results suggest that under conditions of oxygen variation, both shuttles are important, and that iGP1 and AOAA inhibitors should alter growth and mortality of Group 3.

### Inhibition of mGPDH increases the respiratory and glycolytic capacity of Group 3 MB cells

**Fig. 2:**
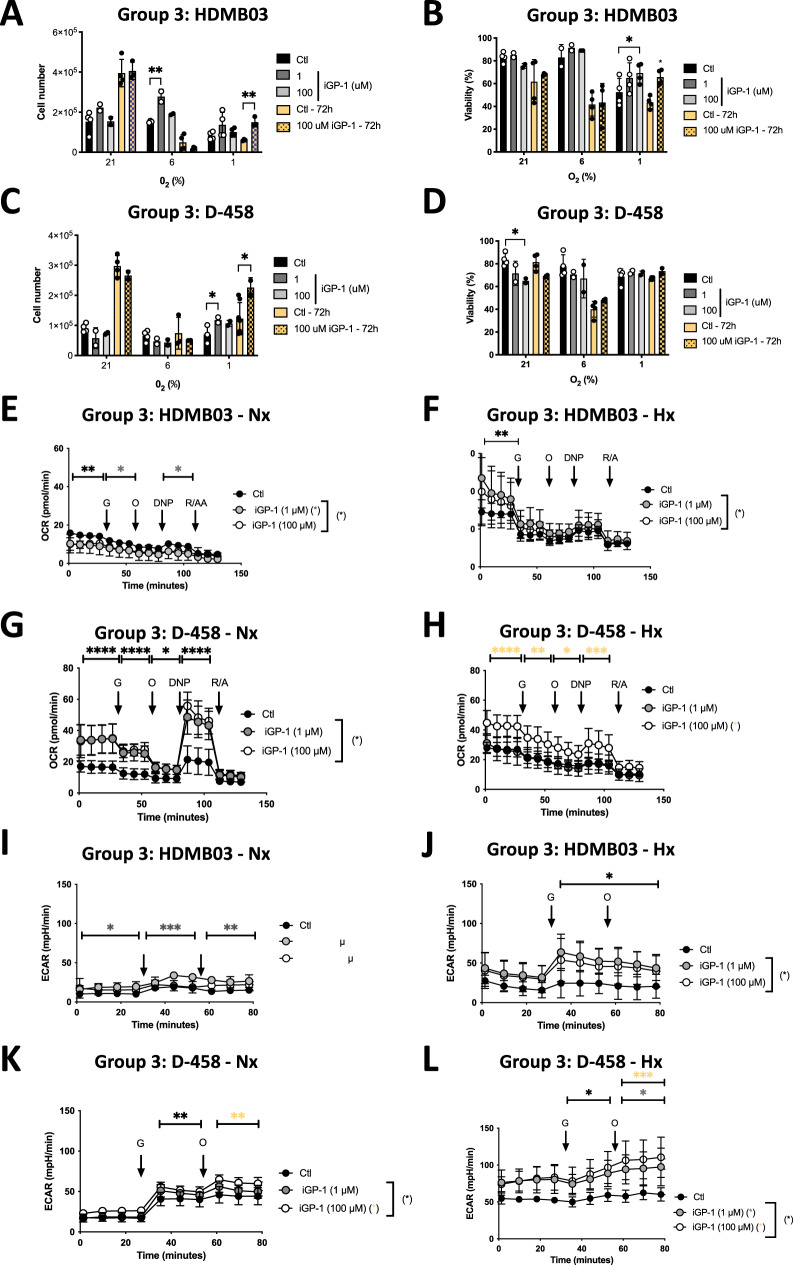
Inhibition of mGPDH by iGP-1 increases OXPHOS and glycolysis in HDMB03 cells (Group 3 MB). HDMB03 (**A**) and D-458 (**C**) cells were seeded at the same density and incubated in 21%, 6%, and 1% O_2_ for 24 h and 72 h in the absence (Ctl) or presence of iGP-1 (1 or 100 µM). Cell proliferation was measured using an ADAM cell counter. HDMB03 (**B**) and D-458 (**D**) cells were seeded at the same density and incubated in 21%, 6%, and 1% O_2_ for 24 h and 72 h in the absence (Ctl) or presence of iGP-1 (1 or 100 µM). Cell viability was measured using an ADAM cell counter. **A**–**D** The 2-way ANOVA is representative of at least three independent experiments. Not significant (ns), *p < 0.05 and **p < 0.005. Respiratory control of HDMB03 cells. OCR was measured in real-time with the XF96 analyzer. Cells were cultured for 24 h in Nx (21% O_2_ - **E**) and Hx (1% O_2_ - **F**) in the absence (Ctl) or presence of iGP-1 (1 or 100 µM). Cells were deprived of glucose for 1 h, then glucose (G), oligomycin (O), DNP, and Rotenone + Antimycin A (R/A) were injected at the indicated times. The graphs are representative of at least three independent experiments carried out in octuplicate. Respiratory control of D-458 cells. OCR was measured in real time with the XF96 analyzer. Cells were cultured for 24 h in Nx (21% O_2_ - **G**) and Hx (1% O_2_ - **H**) in the absence (Ctl) or presence of iGP-1 (1 or 100 µM). Cells were deprived of glucose for 1 h, then glucose (G), oligomycin (O), DNP, and Rotenone + Antimycin A (R/A) were injected at the indicated times. The graphs are representative of at least three independent experiments carried out in octuplicate. ECAR of HDMB03 cells in Nx (21% O_2_ - **I**) and Hx (1% O_2_ - **J**) in the absence (Ctl) or presence of iGP-1 (1 or 100 µM) for 24 h was evaluated with the XF96 analyzer. Cells were deprived of glucose for 1 h, then glucose (G) and oligomycin (O) were injected at the indicated times. The graphs are representative of at least three independent experiments carried out in octuplicate. ECAR of D-458 cells in Nx (21% O_2_ - **K**) and Hx (1% O_2_ - **L**) in the absence (Ctl) or presence of iGP-1 (1 or 100 µM) for 24 h was evaluated with the XF96 analyzer. Cells were deprived of glucose for 1 h, then glucose (G) and oligomycin (O) were injected at the indicated times. The graphs are representative of at least three independent experiments carried out in octuplicate. **E**–**L** Black star (*) represents the statistical differences between iGP1 (1 and 10 µM) and control, gray star (*) between iGP1 (1 µM) and control and orange (*) star between iGP1 (10 µM) and control. The 2-way ANOVA is representative of at least three independent experiments. *p < 0.05, **p < 0.005, ***p < 0.001 and ****p < 0.0001.

Since mGPDH and MDH2 have a strong impact on the metabolism of MB cells under hypoxic conditions, we first examined whether the decrease in mGDPH activity had an impact on the viability, proliferation, and metabolism of the Group 3 MB cells. In the presence of 1 or 100 µM iGP1, at 24 h and 72 h, a significant increase in proliferation was observed with HDMB03 and D-458 (Group 3) in low oxygen concentrations (Fig. 2A, C), in combination with an increase in viability under Hx compared to Nx only for HDMB03 (Fig. 2B). The viability of D-458 was not affected by iGP-1 in Hx (Fig. 2D).

We then hypothesized that the reason for these increases in proliferation and viability observed in the presence of iGP-1 was due to metabolic changes. Thus, we quantified both mitochondrial respiration and glycolysis with the Seahorse XF by measuring the oxygen consumption rate (OCR) and the extracellular acidification rate (ECAR). Interestingly, the overall respiration of Group 3 MB cells (HDMB03 and D-458) in Phx and Hx appeared greater than in Nx (Fig. 2E–H and Supplementary Fig. 3A, B). In parallel, the basal level of glycolysis of Group 3 MB cells (HDMB03 and D-458) was higher in Hx (Fig. 2I–L) and intermediate between Nx and Hx in Phx (Supplementary Fig. S3C, D) and. However, the glycolytic capacity remained unchanged in both HDMB03 and D-458 MB cells.

In the presence of iGP1, the respiratory capacity was almost always increased in both cells, especially under Phx (Fig. 2E–H, Supplementary Fig. S3A-). Glycolytic capacity was increased only in Group 3 cells under Phx and Hx (Fig. 2I–L - Supplementary 3C and D).

Recently, Di Magno et al. proposed a therapeutic potential for phenformin (Phenf) in medulloblastoma in the SHH group [25]. The authors demonstrated that, at clinical doses, Phenf does not act at the level of mitochondrial complex 1 but rather at the level of mitochondrial glycerophosphate dehydrogenase (mGPDH). By acting on mGPDH, Phenf would then attenuate the transfer of reducing equivalents from the cytoplasm to the mitochondria leading to an increase in the lactate/pyruvate ratio and a redox-dependent inhibition of gluconeogenesis from reduced but non-oxidized substrates.

To better identify the dual function of Phenf (mitochondrial complex 1 and/or mGPDH blockade) on Group 3 cells, we compared its action in Hx on cell metabolism, proliferation, and viability. We also used Rotenone, a potent and specific inhibitor of mitochondrial complex 1, as a control. Cells were challenged with Phenformin (Phenf – 3 µM and 100 µM) and Rotenone (2.5 µM). In HDMB03 cells, Rotenone was less potent to block the respiration compared to Phenf 3 µM and 100 µM (Supplementary 4A). Rotenone and Phenf (3 µM and 100 µM) blocked similarly the respiration in D-458 cells (Supplementary Fig. 4B). We examined the cumulative effect of both Phenf and iGP-1 to determine if Phenf could compensate for the sublimating effects of iGP1 on respiration and thus allow mGPDH to be fully blocked without further counterparts. While proliferation was greatly reduced with Phenf alone or Phenf+iGP-1, HDMB03 MB cells presented a decrease in viability with both Phenf or Phenf+iGP-1 (Supplementary Fig. 5SA, B). The Phenf+iGP1 combo better sensitized HDMB03 to cell death under hypoxic conditions, with HDMB03 dying twice as much. D-458 cells were more resistant to the combo (Supplementary Fig. S5C, D). These results clearly show that Phenformin, at low or usual concentration, has a direct action on mitochondrial respiration in Group 3 cells, regardless of the concentration used like Rotenone, confirming its involvement at the level of mitochondrial complex I. However, while Phenf and iGP1 may have a similar target, mGPDH, the consequences of iGP-1 actions are different from those of Phenformin, one blocking respiration, the other inducing it.

We then hypothesized that reactive oxygen species (ROS), generated by mGPDH [26], may be inhibited by iGP1, thus promoting high viability and better proliferation under low oxygen conditions. In the presence of the ROS inhibitor N-Acetyl Cysteine (NAC), HDMB03 and D-458 cells showed higher viability: 72.5 ± 4.8 versus 61.5 ± 7.05 and 76.3 ± 4.7 versus 51.8 ± 5.1 respectively (Supplementary Fig. S6). On the other hand, the presence of NAC in combination with iGP1 was not able to increase cell viability, strongly supporting the ROS inhibitory role of iGP1.

Taken together, these results showed that mGPDH inhibition by iGP1 allows Group 3 MB cells to switch to glycolysis, thereby increasing their cell growth. Although cells from Group 3 oxidize G3P via mGDPH under Phx or Hx, this activity tends to stunt the cells in their development, probably due to ROS production. The results demonstrate that blocking mGPDH with iGP1 as the only inhibitor cannot be used to specifically kill Group 3 MB cells but should be combined with another inhibitor of a similar nature, a specific respiratory inhibitor such as Phenf.

### Group 3 MB cells rely on both Malate-Aspartate shuttle (MAS) and mGPDH shuttle in Hx

**Fig. 3:**
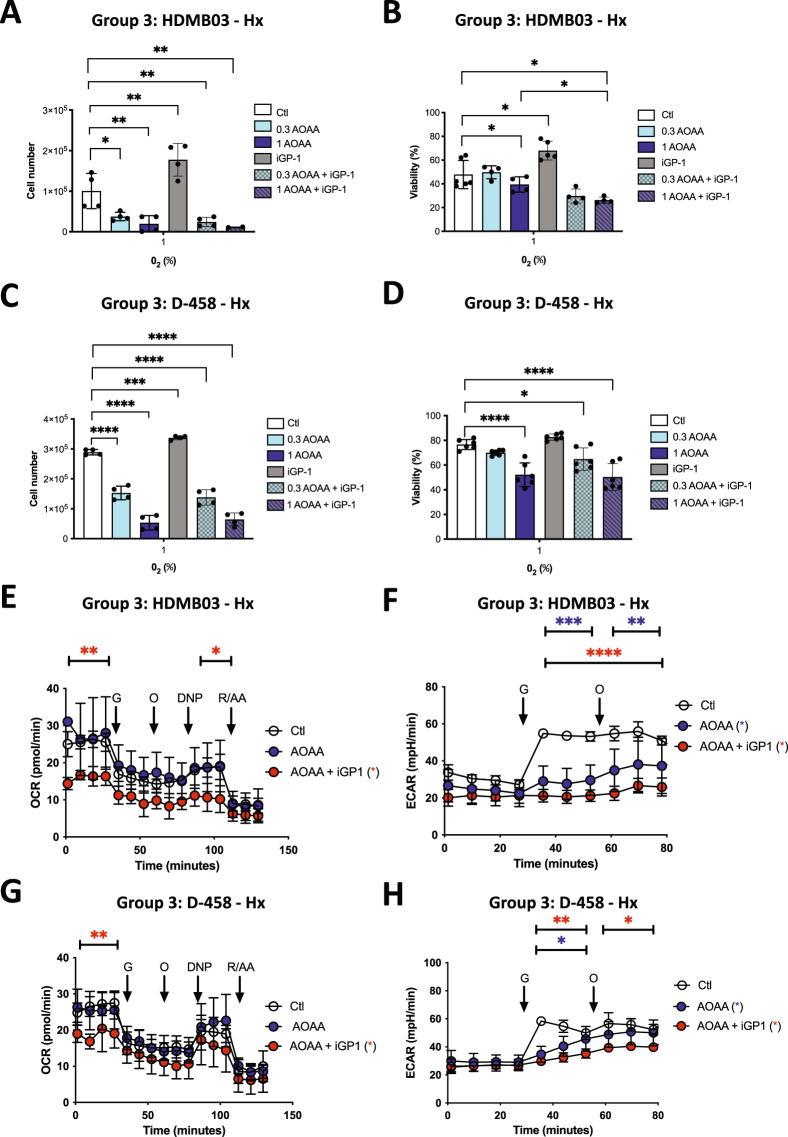
Group 3 cells rely on both G3PS and MAS shuttles under Hx. HDMB03 (**A**) and D-458 (**C**) cells were seeded at the same density and incubated in 1% O_2_ for 72 h in the absence (Ctl) or presence of AOAA (0.3 and 1 mM), iGP-1 (100 µM) or AOAA+iGP-1. Cell proliferation was measured using an ADAM cell counter. HDMB03 (**B**) and D-458 (**D**) cells were seeded at the same density and incubated in 1% O_2_ for 72 h in the absence (Ctl) or presence of AOAA (0.3 and 1 mM), iGP-1 (100 µM) or AOAA+iGP-1. **A**–**D** The ordinary one-way ANOVA is representative of at least four independent experiments. Not significant (ns), *p < 0.05, **p < 0.006, ***p = 0.001 and ****p < 0.0001. HDMB03 (**E**) and D-458 (**G**) cells. OCR was measured in real time with the XF96 analyzer. Cells were cultured for 24 h in Hx (1% O_2_) in the absence (Ctl) or presence of AOAA (1 mM) or AOAA+iGP-1 (100 µM). Cells were deprived of glucose for 1 h, then glucose (G), oligomycin (O), DNP, and Rotenone + Antimycin A (R/A) were injected at the indicated times. The graphs are representative of at least three independent experiments carried out in octuplicate. ECAR in Hx (1% O_2_) in the absence (Ctl) or presence of AOAA (1 mM) or AOAA+iGP-1 (100 µM) for 24 h of D-458 (**F**) and HDMB03 (**H**) cells was evaluated with the XF96 analyzer. Cells were deprived of glucose for 1 h, then glucose (G) and oligomycin (O) were injected at the indicated times. The graphs are representative of at least three independent experiments carried out in octuplicate. **E**–**H** Blue star (*) represents the statistical differences between AOAA and control, red star (*) between AOAA + iGP1. The 2-way ANOVA is representative of at least three independent experiments. *p < 0.05, **p < 0.005 and ***p < 0.001.

We used the MAS inhibitor, AOAA, to block mMDH activity in both cell groups. We also combined the two inhibitors iGP1 and AOAA in Hx to totally block both the MAS and mGPDH shuttles. All cells showed sensitivity to 1 mM AOAA in both proliferation and viability (Fig. 3A–D) suggesting that cells from Group 3 MB use the MAS shuttle equivalently. The AOAA+iGP-1 combination did show a slight additive effect on decreasing viability in HDBM03 and D-458 MB cells (Fig. 3B, D).

Respiration analysis showed that the combination AOAA+iGP-1 was required to block mitochondrial respiration in Group 3 MB cells (Fig. 3E, G) and also decreased the glycolytic capacity of Group 3 MB cells (Fig. 3F, H).

These results indicate that the functioning of Group 3 cells relies on both shuttles since both inhibitors were needed to observe an inhibition effect, suggesting a high dependency on NAD+/NADH metabolism for Group 3 MB cells.

### Inhibition of LDHA in combination with iGP1 and AOAA massively kills all Group 3 cells under Hx

We hypothesized that the other NAD+ production pathway generated by lactate dehydrogenase could be used when both shuttles are blocked and therefore addressed blocking it as well.

**Fig. 4:**
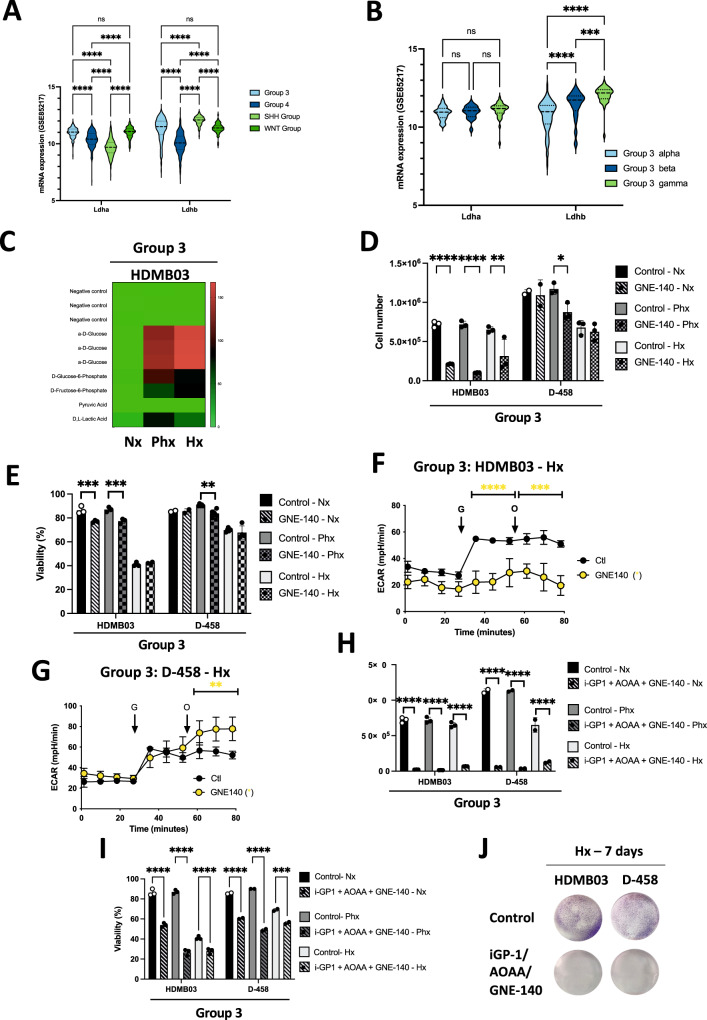
Inhibition of LDHA in combination with iGP1 and AOAA massively kills Group 3 MB cells. **A** Heatmap of the RNA expression of *GPDH2* and *MDH2* genes from GSE85217 comprising 763 primary samples from MB patients from Group 3, Group 4, SHH Group and WNT Group MB [19]. Expression of the genes was compared using Phantasus (v1.19.3). **B** Violin plot of the RNA expression of *LDHA* and *LDHB* genes from GSE85217 comprising 763 primary samples from MB patients from Group 3 alpha, Group 3 beta and Group 3 gamma MB [19]. Expression of the genes was compared using Prism 9 version 9.5.0. **A**, **B** The 2-way ANOVA is used to determine statistically difference between the different groups. Not significant (ns), ***p = 0.0001 and ****p < 0.0001. **C** Heatmap showing the seven substrates that were differently metabolized by HDMB03 and ONS-76 in Nx, Phx and Hx. The color key scale for each substrate is based on dye reduction quantified by Omnilog units. A dark red color indicates strong positive substrate metabolization, a red color moderate metabolization and a green color indicates no substrate metabolization. **D**, **E** HDMB03 and D-458 cells were seeded at the same density and incubated in Nx, Phx and Hx for 72 h in the absence (Ctl) or presence of GNE-140 (5 µM). Cell proliferation (**C**) and viability (**D**) were measured using an ADAM cell counter. **D** and **E** The 2-way ANOVA is representative of at least three independent experiments. Not significant (ns), *p = 0.014, **p < 0.005, ***p < 0.001 and ****p < 0.0001. ECAR in Hx (1% O_2_) in the absence (Ctl) or presence of GNE-140 (5 µM) for 24 h of HDMB03 (**F**) and D-458 (**G**) cells was evaluated with the XF96 analyzer. Cells were deprived of glucose for 1 h, then glucose (G) and oligomycin (O) were injected at the indicated times. The graphs are representative of at least three independent experiments carried out in octuplicate. Yellow star (*) represents the statistical differences between GNE-140 and control. **H**, **I** HDMB03 (**H**) and D-458 (**I**) cells were seeded at the same density and incubated in Nx, Phx and Hx for 72 h in the absence (Ctl) or presence of iGP-1 (100 µM) + AOAA (1 mM) + GNE-140 (5 µM). Cell proliferation (**H**) and viability (**I**) were measured using an ADAM cell counter. **H** and **I** The 2-way ANOVA is representative of at least three independent experiments. ***p < 0.001 and ****p < 0.0001. **J** Clonogenic assay of HDMB03 and D-458 cells. Cell lines were seeded at the same density and incubated in Hx (1% O_2_) for 7 days in the absence (Ctl) or presence of iGP-1 (100 µM) + AOAA (1 mM) + GNE-140 (5 µM).

From the Affymetrix Human Gene 1.1 ST array profiling of 763 primary medulloblastoma samples (GSE85217) [19], *LDHA* and *LDHB* gene expressions were different within the 4 groups (Fig. 4A). Gene expression of *LDHA* in Group 3 was significantly higher compared to Groups 4 and SHH, whereas *LDHB* gene expression was higher in Group 3 compared to Group 4 but lower compared to the SHH Group. Interestingly, *LDHA* expression was homogenous in the three subtypes of Group 3 but *LDHB* gene expression was significantly lower in Group 3 alpha compared with both beta and gamma (Fig. 4B). These results suggested strong heterogeneity in the expression of both *LDHA* and *LDHB* genes, both at the group and subtype levels. These results are also in favor of a possible axis for NAD+ formation.

We therefore used GNE-140, which is a potent inhibitor of LDHA, to block the transformation of pyruvate to lactate. It also blocks LDHB, which allows the reverse transformation. The results obtained with Omnilog clearly showed that all cells, regardless of group, were avid for glucose but also for glucose-6 phosphate and Fructose-6 phosphate (Fig. 4C). This ability to grow on these compounds was stronger under Phx and Hx conditions for HDMB03 cells compared to Nx. Group 3 MB cells showed different sensitivities to GNE-140 in term of proliferation (Fig. 4D) and viability (Fig. 4E), with HDMB03 cells appearing more sensitive. Basal glycolysis and glycolytic capacity were decreased in HDMB03 MB cells in the presence of GNE-140 (Fig. 4F), while there was no impact on either basal glycolysis or glycolytic capacity in D-458 MB cells, which would explain why these cells can grow in the presence of GNE-140 in hypoxia (Fig. 4G).

Finally, we tested GNE-140 with the different combinations previously used, to block any potential for NAD+ production and any switch to lactate production. The addition of GNE-140 clearly potentiated the effects of the AOAA+iGP-1 combo (Fig. 4H, I), regardless of the cell line used. The iGP-1 + AOAA + GNE-140 combo massively reduced the proliferation and/or killed the Group 3 MB cells, since the few colonies visible in Group 3 did not grow (Fig. 4J).

These results strongly suggest that i) Group 3 MB cells are definitely very sensitive when one or more metabolic pathways are blocked in hypoxia, ii) the addition of GNE-140 strongly sensitizes all cell lines, even the most refractory ones such as D-458 Group 3 MB cells, and finally iii) we have characterized new therapeutic leads to potentially target the most treatment-refractory cells (Group 3 MB).

### Blocking mitochondrial NADH shuttles and NAD+ recycling induces apoptosis of Group 3 tumor MB organoids

**Fig. 5:**
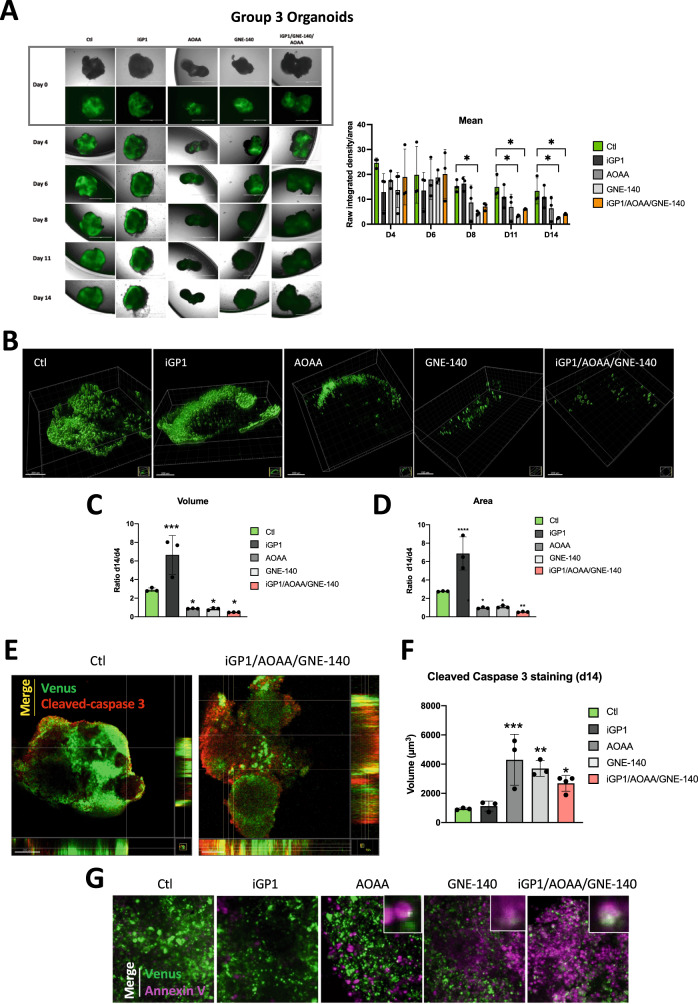
Validation of drug response in human Group 3 MB tumor organoids. **A** Brightfield and fluorescence images of cerebellar organoids at day 71 (Day 0 of treatment), day 75 (Day 4 of treatment), day 77 (Day 6 of treatment), day 79 (Day 8 of treatment), day 82 (Day 11 of treatment) and day 85 (Day 14 of treatment) electroporated at day 35 with pBase + pPBMYC + pPBOtx2 + pPBVenus. Right panel: Quantification of the mean (raw integrated density/area using Fiji) for each condition from day 4 (D4) to day 14 (D14). The ordinary one-way ANOVA is representative of at least three independent organoids. *p < 0.05. **B** Three-dimensional structures at day 14 obtained from confocal image series using IMARIS software; scale bars = 200 µm. Quantification of cell volume (**C**), and area (**D**) at day 4 and day 14. A ratio was calculated from day 14 to day 4. **C**, **D** The 2-way ANOVA is representative of at least three independent experiments. Not significant (ns), *p = 0.014, **p < 0.005, ***p < 0.001 and ****p < 0.0001. **E** Superimposition images where the presence of both fluors, Venus (tumor cells) and red (cleaved- caspase 3) is shown as a third color (yellow - merge). Human Group 3 MB tumor organoids have been treated in the absence (Ctl) or presence (iGP1/AOAA/GNE-140) of the combo. The orthogonal view is used to show virtual cross sections—one plotted along the x-axis and the other plotted along the y-axis; scale bars = 100 µm. **F** Quantification of cell volume day 14 after staining with cleaved caspase 3. The ordinary one-way ANOVA is representative of at least three independent experiments. *p < 0.05, **p < 0.005 and ***p < 0.001. **G** Superimposition images with the confocal (x20) where the presence of both fluors, Venus (tumor cells) and magenta (annexin V – Far red) is shown as a third color (white – merge) Human Group 3 MB organoids have been treated in the absence (Ctl) or presence of iGP-1, AOAA, GNE-140 and iGP1/AOAA/GNE-14.

Finally, we sought to validate the drug responses identified in 2D on an organoid-derived model of human Group 3 MB in which cancer cells are GFP/Venus positive [22]. Human Group 3 MB organoids have been generated with MYC and Otx2 overexpression, to recapitulate gene alteration/expression of Group3 MB patients. We examined three single drugs (iGP1, AOAA, and GNE-140) and a combination of drugs (iGP1+GNE-140 + AOAA). The organoids were maintained for 14 days and treated every 4 days with the different compounds (Fig. 5A). We observed significant antitumor activity of GNE140 at day 8. This effect, and the drug combo, continued to day 11 and the Venus fluorescence disappeared almost completely by day 14, indicating a nearly total disappearance of the tumor cells represented by the calculation of the mean (raw integrated density/area). AOAA showed only a tendency from day 8 to day 14. Confocal image stacks of the tumoral Venus cells were reconstructed to 3D isosurfaces (Imaris) (Fig. 5B). The volume (Fig. 5C), and area (Fig. 5D) showed significant decreases compared to the control. Our results confirmed that, in the presence of iGP1, tumor cells proliferate better, similar to what we obtained in 2D. Finally, control cells showed less cleaved caspase 3 staining than cells in the presence of iGP1+GNE-140 + AOAA (Fig. 5E). Moreover, super imposition of images double stained with green (Venus) and red (cleaved caspase 3) fluors showed that tumor cells expressed both proteins, strongly suggested that cells died of apoptosis in the presence of the drug combo but also in the presence of the other compounds alone (Fig. 5F). These results were also confirmed with annexin V, commonly used to detect apoptosis (Fig. 5G).

These findings suggest that the drug responses observed in 2D can be recapitulated when assayed with the human Group 3 MB tumor organoids. Interestingly, we found that AOAA, to a less extend, and GNE140, two specific inhibitors of NAD+ production, rapidly reduce the number of tumor cells. The drug combo, certainly more drastic for the cell, is also clearly effective.

## Discussion

Our study studies Group 3 (HDMB03 and D-458) MB cells in a laboratory setting of 21% O_2_, an estimated physiological setting of 6% O_2_, and a tumor pathophysiological setting of 1% O_2_. While Bernauer et al.’s review [27] clearly explains the importance of hypoxia in cancer cell resistance, our article addresses the issue of metabolism in a hypoxic environment and its therapeutic possibilities. We bring considerable expertise and experience from many years of work using this approach in colon [28, 29] and kidney [30] cancers and focus on the critical role of mitochondria [31, 32].

Our results clearly show differences between 21% and 6% O_2_ and between 6% and 1% O_2_, for example the impact of iGP-1 on respiration (OCR) and glycolysis (ECAR), respectively (Fig. 2 and Supplementary Fig. 3). Different conclusions on the impact of iGP1 would be drawn depending on whether the experiments were done at 21 or 6%. We also observed that it was more difficult to reproduce our experiments identically at 6% O_2_ compared to 21% or 1% and that we very often had large standard deviations. It appeared to us that the cells could be torn between two worlds, that of normoxia in which the cell lines have always been artificially immersed in vitro and that of hypoxia where their natural tumor metabolism is better able to express itself. The conditions at 6% O_2_ are certainly not linear conditions intermediate between 21% and 1%. These are the closest conditions to those in which the normal cell must live and adapt in the brain [17, 18]. The tumor cell will then be confronted with a much larger O_2_ gradient under less fixed and much more dynamic conditions. Thus, our decision to examine a wide range of O_2_ concentrations, focusing primarily on hypoxia, where tumor cells are most resistant to therapies, and then move to organoids, a model in which a more realistic environmental heterogeneity can begin to form. It therefore seems interesting to target metabolic pathways in their microenvironmental context where hypoxia is strongly involved.

Because of the dependence of cancer cells on the glycolysis-oxidative phosphorylation shunt, proteins that contribute to the glycolysis-OXPHOS link may be promising anticancer therapeutic targets. In this sense, the pathways of NAD+ regeneration through the malate/aspartate and glycerol-phosphate shuttles have proven to be extremely interesting. The two shuttles, G3PS and MAS, in our study, clearly showed similar sensitivity to hypoxia. First, the mRNA of mGPDH and MDH2 in Group 3 cells do not seem to be affected by the oxygen variations used whereas in HDMB03 cells it is mainly at the level of protein expression. Group 3 cells always responded strongly to the metabolic inhibitors (iGP1, AOAA, GNE-140, Phenf). We have thus clearly highlighted the Achilles heel of the Group 3 MB cells: the hypoxic metabolism. If blocking mGPDH alone did not appear to be the best therapeutic approach, blocking MAS with AOAA or concomitantly with mGPDH seems to be a very promising approach confirmed by the use on human Group 3 MB tumor organoids. We note that AOAA has already been administered in trials relating to Huntington’s disease [33] and tinnitus, and that toxic side effects and dosage are issues to be addressed.

Similarly, GNE-140, the LDHA/LDHB inhibitor, was also a very good candidate for Group 3 MB cells. Significant evidence exists to support the development of LDH inhibitors as a therapeutic option for cancer treatment [34–38], but we did not find any ongoing clinical trials with this drug currently. The relatively poor pharmacokinetics of this compound have limited its usefulness for testing in vivo. However, we have clearly shown that its action appears to be effective and rapid in the human Group 3 MB organoid model.

Finally, our results support previous findings that a 3D model presents heterogeneous conditions of normoxia, physioxia and hypoxia, with kinetics closer to pathological conditions than a 2D model. This coculture method better reflects the tumor microenvironment and provides a good platform for screening and testing treatment plans. It would be worthwhile testing our different drugs in conjunction with current radio- and chemo-therapies (Cyclophosphamide, Gemcitabine, Axitinib or Galunisertib) [39, 40], but also to follow the fate of the tumor cells after the treatments are stopped.

Organoids thus appear to be a promising tool, paving the way for valuable advances in the development of new treatments for Group 3 patients with high levels of MYC and Otx2.

### Supplementary information

Suppl. Figures Legends

aj-checklist

Uncropped Western blots

Suppl. Figure 1

Suppl. Figure 2

Suppl. Figure 3

Suppl. Figure 4

Suppl. Figure 5

Suppl. Figure 6

## Data Availability

All datasets generated and analyzed during this study are included in this published article and its Supplementary Information files. Additional data are available from the corresponding author on reasonable request.
