# Supplementary material for: The mitochondrial NADH shuttle system is a targetable vulnerability for Group 3 medulloblastoma in a hypoxic microenvironment
Source: Cell Death Dis. 2023 Nov 30;14(11):784. doi: 10.1038/s41419-023-06275-0 (PMC10689432; doi:10.1038/s41419-023-06275-0)
Supplement: Supplementary file 1 — Suppl. Figures Legends [file 41419_2023_6275_MOESM1_ESM.doc]

**Supplementary legends**

**Supplemental Figure 1:** **A** Graphic representation of *mGPDH* mRNA expression in HDMB03 and D-458 cells incubated in normoxia (Nx) for 24h and hypoxia (Hx - 1% O_2_) for 24h, 48h and 72h. **B** Graphic representation of *MDH2* mRNA expression in HDMB03 and D-458 cells incubated in normoxia (Nx) for 24h and hypoxia (Hx - 1% O_2_) for 24h, 48h and 72h. **C** HDMB03 and D-458 cells were transfected with a siRNA control (siCtl) or siRNA targeting mGPDH (simGPDH) for 24h. Cells were then incubated in normoxia (Nx), physioxia (Phx) and hypoxia (Hx) for 72h. Cell lysates were analyzed by immunoblotting for mGPDH. Hsp90 was used as a loading control. Histogram of densitometric analysis represented on the bottom panel. The 2-way ANOVA is representative of at least two independent experiments. Not significant (ns), * p=0.0292, ** p=0.0019 and **** p<0.0001. **D** HDMB03 and D-458 cells were transfected with a siRNA control (siCtl) or siRNA targeting MDH2 (siMDH2) for 24h. Cells were then incubated in normoxia (Nx), physioxia (Phx) and hypoxia (Hx) for 72h. Cell lysates were analyzed by immunoblotting for MDH2. Hsp90 was used as a loading control. Histogram of densitometric analysis represented on the bottom panel. The 2-way ANOVA is representative of at least two independent experiments. ** p<0.005 and *** p<0.0005.

**Supplemental Figure 2:** **A** Heatmap showing the Glycerol 3P substrate that was differently metabolized by Group 3 MB cells (HDMB03 and D-458) in Nx, Phx and Hx. The color key scale for each substrate is based on dye reduction quantified by Omnilog units. A dark red color indicates strong positive substrate metabolization, a red color moderate metabolization and a green color indicates no substrate metabolization. **B** Heatmap showing the nine substrates or combination of substrates that were differently metabolized by Group 3 MB cells (HDMB03 and D-458) in Nx, Phx and Hx. The color key scale for each substrate is based on dye reduction quantified by Omnilog units. A dark red color indicates strong positive substrate metabolization, a red color moderate metabolization and a green color indicates no substrate metabolization. **C** HDMB03 cells were transfected with siRNA Control (siCtl), siRNA targeting mGPDH (simGPDH – 100nM) or treated with iGP-1 (10 or 100µM) for 72h in Hx. G3P was then detected by colorimetric assay. The ordinary one-way ANOVA is representative of at least three independent experiments. * p<0.01 and *** p=0.0002. **D** HDMB03 cells were treated with iGP-1 (100µM) or transfected with siRNA Control (siCtl), or siRNA targeting mGPDH (simGPDH – 100nM) for 72h in Hx. The ratio of NAD/NADH was then quantified by colorimetric assay. The ordinary one-way ANOVA is representative of at least three independent experiments. **** p<0.0001. **E** HDMB03 cells were transfected with siRNA Control (siCtl), siRNA targeting MDH2 (siMDH2) or treated with AOAA (1mM) for 72h in Hx. Malate concentration was then detected by colorimetric assay. The ordinary one-way ANOVA is representative of at least three independent experiments. * p=0.0212. **F** Metabolites differentiating between control (Ctl) and iGP-1 or AOAA HDMB03 treated cells in Hx (VIP>1.0, metabolites with VIP>1.5 are shown). Relative metabolite abundance is indicated in the bar, with red representing metabolite accumulation.

**Supplemental Figure 3:** **A-C** Respiratory control of HDMB03 (**A**) and D-458 (**C**) cells. OCR was measured in real time with the XF96 analyzer. Cells were cultured for 24h in Phx (6% O_2_) in the absence (Ctl) or presence of iGP-1 (1 or 100 µM). Cells were deprived of glucose for 1h, then glucose (G), oligomycin (O), DNP, and Rotenone + Antimycin A (R/A) were injected at the indicated times. The graphs are representative of at least three independent experiments carried out in octuplicate. **B**-**D**, ECAR of HDMB03 (**B**) and D-458 (**D**) cells in Phx (6% O_2_) in the absence (Ctl) or presence of iGP-1 (1 or 100µM) for 24h was evaluated with the XF96 analyzer. Cells were deprived of glucose for 1h, then glucose (G) and oligomycin (O) were injected at the indicated times. The graphs are representative of at least three independent experiments carried out in octuplicate. The 2-way ANOVA is representative of at least three independent experiments. **A-D** Black star (*) represents the statistical differences between iGP1 (1 and 10 µM) and control, grey star (*) between iGP1 (1µM) and control, orange star (*) between iGP1 (100µM) and control. The 2-way ANOVA is representative of at least three independent experiments. * p<0.05, ** p<0.005, *** p< 0.001 and **** p<0.0001.

**Supplemental Figure 4:** **A-B,** Respiratory control of HDMB03 (**A**) and D-458 (**B**) cells in Hx. OCR was measured in real time with the XF96 analyzer. Cells were cultured for 24h in Phx (6% O_2_) in the absence (Ctl) or presence of iGP-1 (1 or 100 µM). Cells were deprived of glucose for 1h, then glucose (G), oligomycin (O), DNP, and Rotenone + Antimycin A (R/A) were injected at the indicated times. The graphs are representative of at least three independent experiments carried out in octuplicate. The 2-way ANOVA is representative of at least three independent experiments. **A and D** Black star (*) represents the statistical differences between Phenf (3 and 100 µM)/Rotenone and control, orange star (*) between Phenf (3µM) and control, grey star (*) between Phenf (100µM) and control, and green star (*) between Rotenone and control. The 2-way ANOVA is representative of at least three independent experiments. * p<0.05, ** p< 0.005 and ****p< 0.0001.

**Supplemental Figure 5:** iGP1 and Phenf combo induces high cell death in HDMB03 cells (Group 3 MB). **A** and **C**, HDMB03 (**A**) and D-458 (**C**) cells were seeded at the same density and incubated in 21%, 6% and 1% O_2_ for 72h in the absence (Ctl) or presence of iGP-1 (100µM), Phenf (100µM) or Phenf+iGP-1. Cell proliferation was measured using an ADAM cell counter. The 2-way ANOVA is representative of at least two independent experiments. * p<0.05, ** p=0.005, *** p=0.0002 and **** p<0.0001. **B** and **D**, HDMB03 (**B**) and D-458 (**D**) were seeded at the same density and incubated in 21%, 6% and 1%% O_2_ for 72h in the absence (Ctl) or presence of iGP-1 (100µM), Phenf (100µM) or Phenf+iGP-1. Cell viability was measured using an ADAM cell counter. The 2-way ANOVA is representative of at least two independent experiments. The 2-way ANOVA is representative of at least three independent experiments. * p<0.05, ** p<0.005, and **** p<0.0001.

**Supplemental Figure 6:** HDMB03 and D-458 cells were seeded at the same density and incubated in 1% O_2_ for 72h in the absence (Ctl) or presence of NAC, iGP-1 (100µM) or NAC + iGP-1. Cell viability was measured using an ADAM cell counter. The 2-way ANOVA is representative of at least three independent experiments. * p=0.0373, ** p=0.0086, and **** p<0.0001.
