# Supplementary figures and images for: The mitochondrial NADH shuttle system is a targetable vulnerability for Group 3 medulloblastoma in a hypoxic microenvironment
Source: Cell Death Dis. 2023 Nov 30;14(11):784. doi: 10.1038/s41419-023-06275-0 (PMC10689432; doi:10.1038/s41419-023-06275-0)

Suppl. Figure 1C

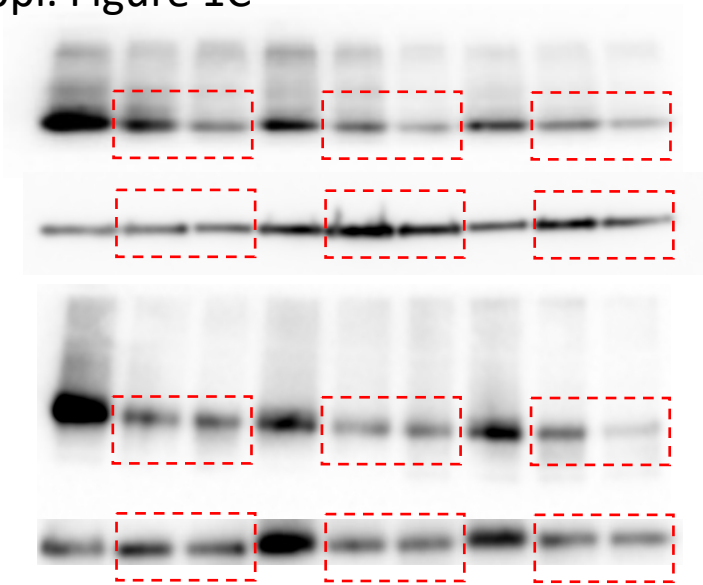

Suppl. Figure 1D

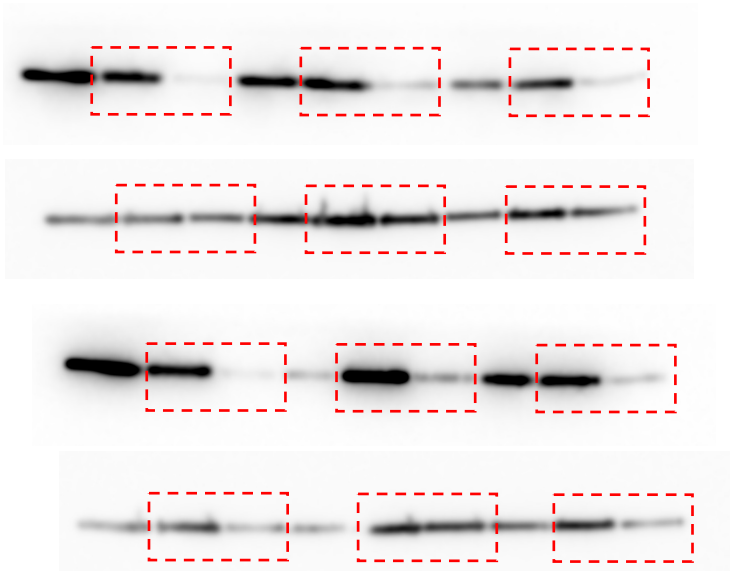

Supplement: Supplementary file 3 — Uncropped Western blots [file 41419_2023_6275_MOESM3_ESM.pdf]

**A**

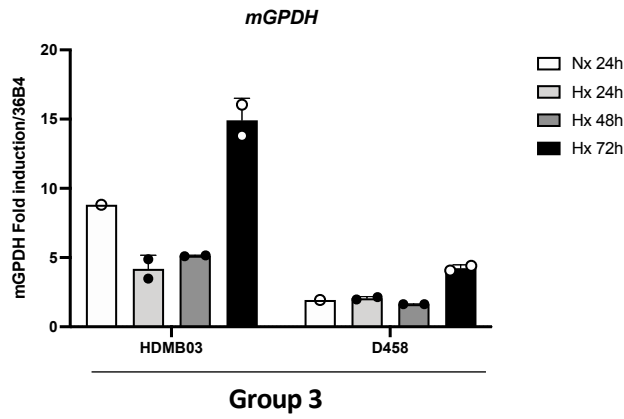

**B**

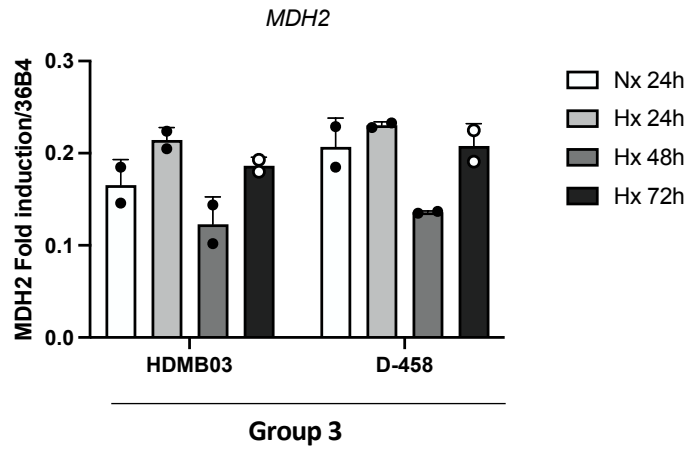

**C**

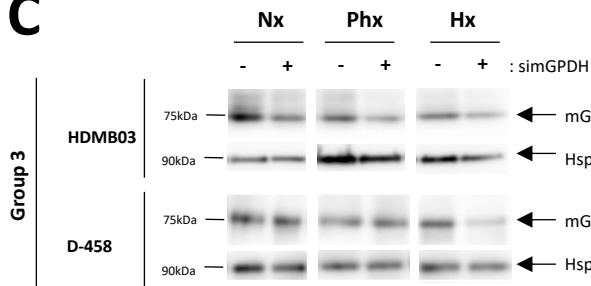

*mGPDH*

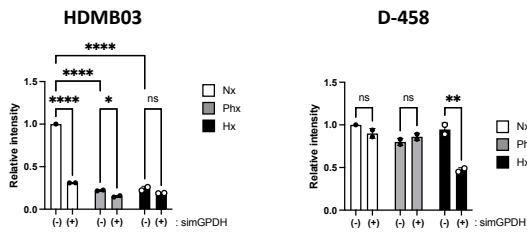

**D**

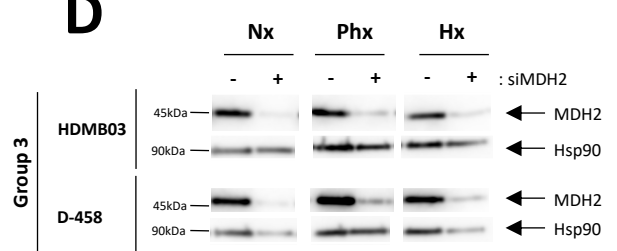

*MDH2*

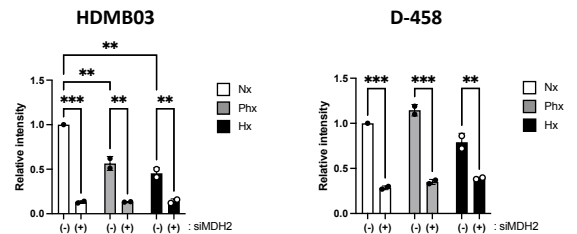

Supplement: Supplementary file 4 — Suppl. Figure 1 [file 41419_2023_6275_MOESM4_ESM.pdf]

A

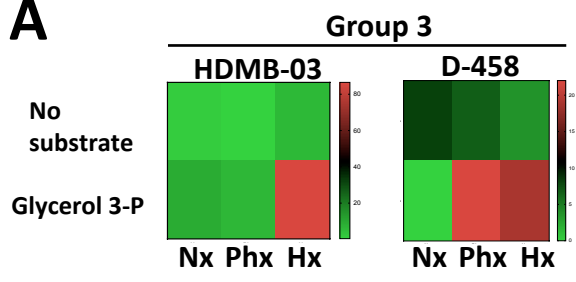

B

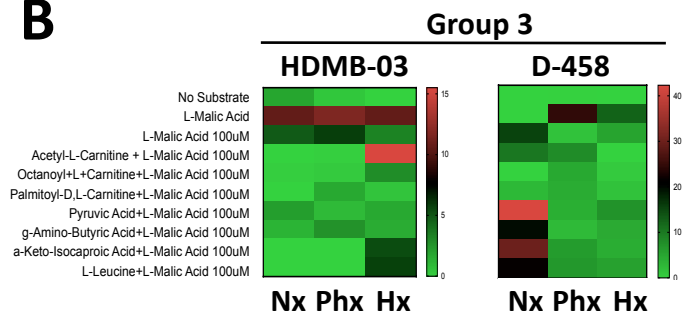

C

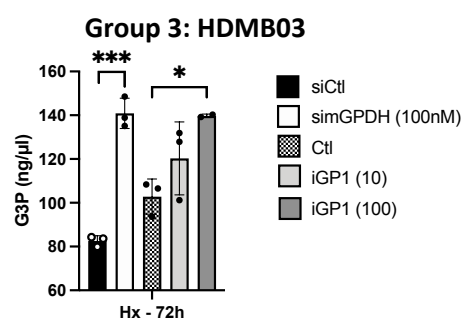

D

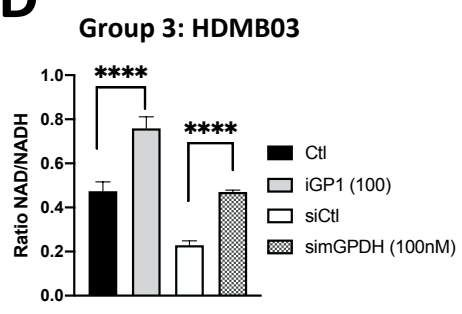

E

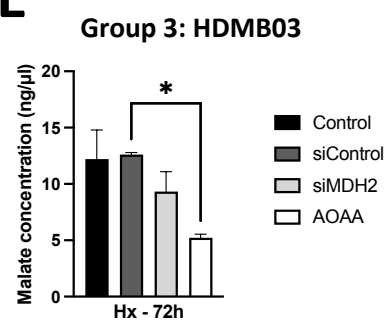

F

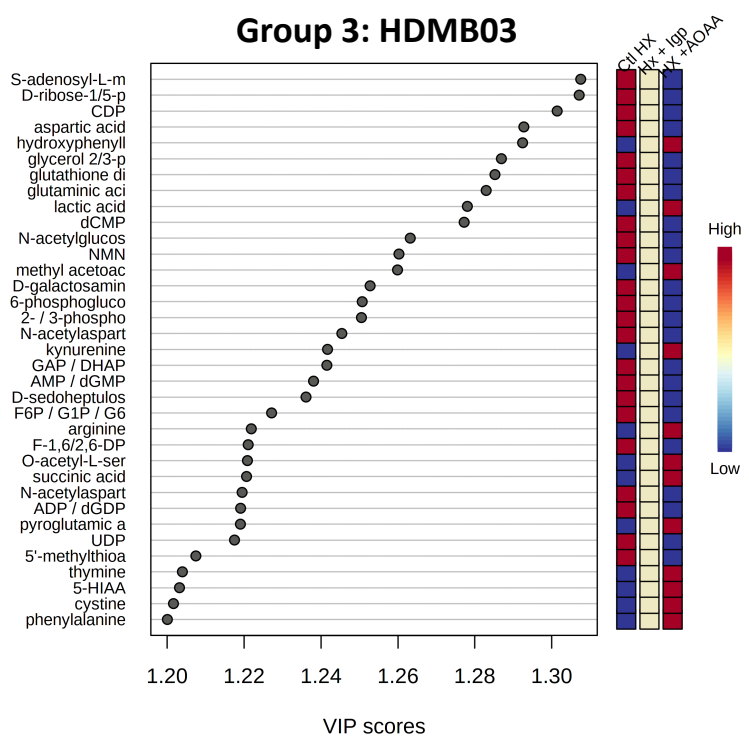

Supplement: Supplementary file 5 — Suppl. Figure 2 [file 41419_2023_6275_MOESM5_ESM.pdf]

**A****Group 3: HDMB03 - Phx**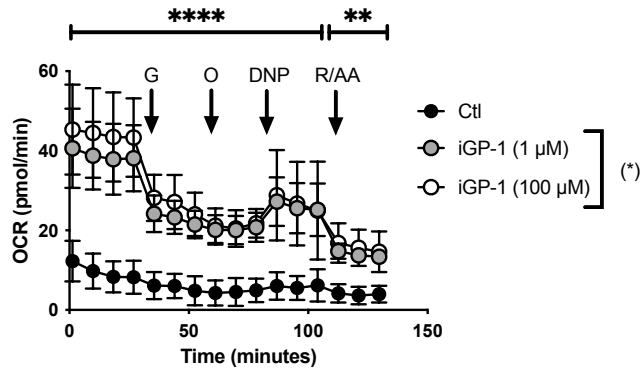**B****Group 3: HDMB03 - Phx**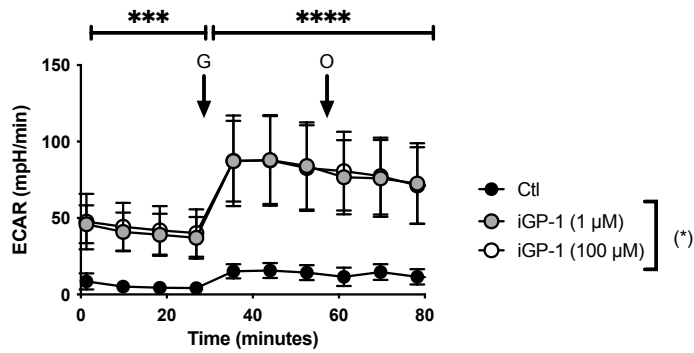**C****Group 3: D-458 - Phx**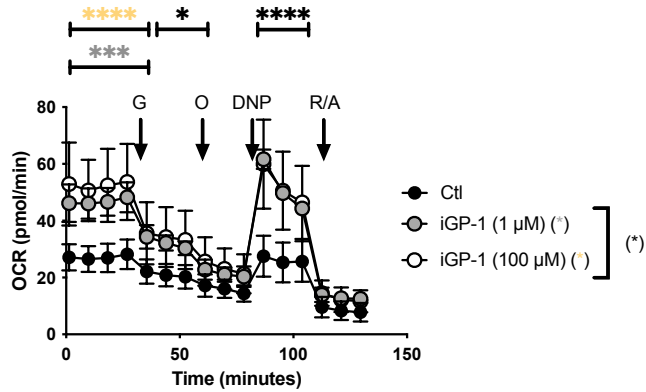**D****Group 3: D-458 - Phx**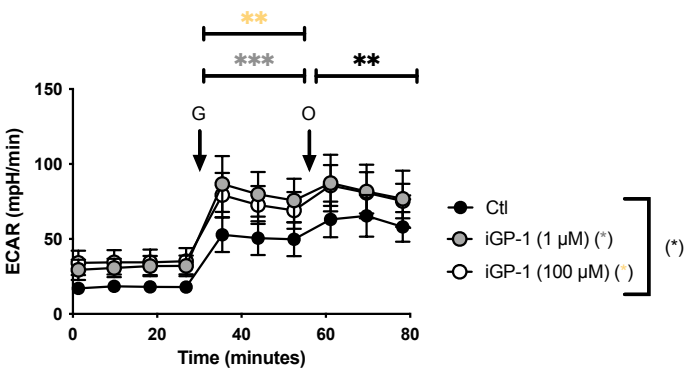

Supplement: Supplementary file 6 — Suppl. Figure 3 [file 41419_2023_6275_MOESM6_ESM.pdf]

**A**

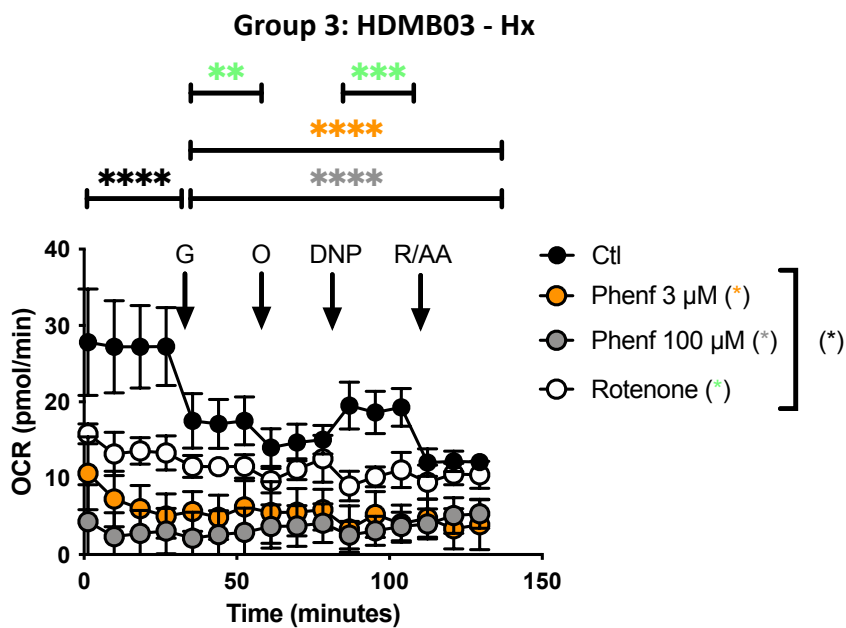

**B**

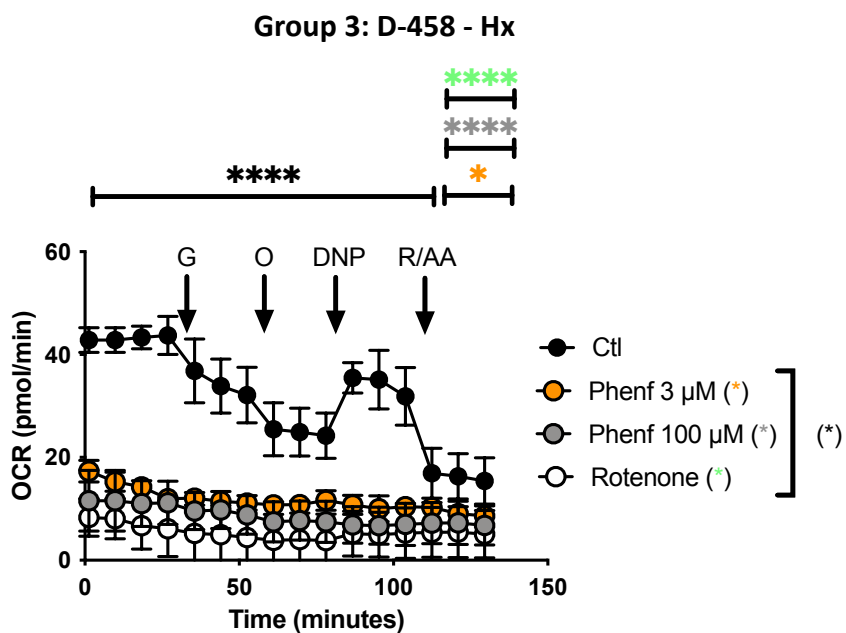

Supplement: Supplementary file 7 — Suppl. Figure 4 [file 41419_2023_6275_MOESM7_ESM.pdf]

**A****Group 3: HDMB03**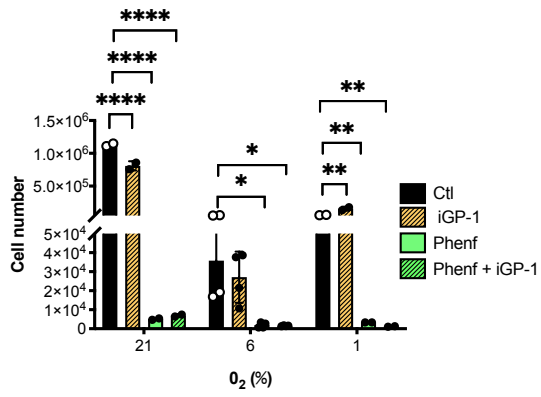**B****Group 3: HDMB03**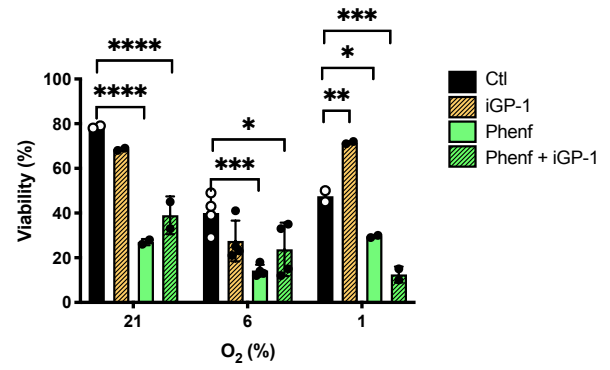**C****Group 3: D-458**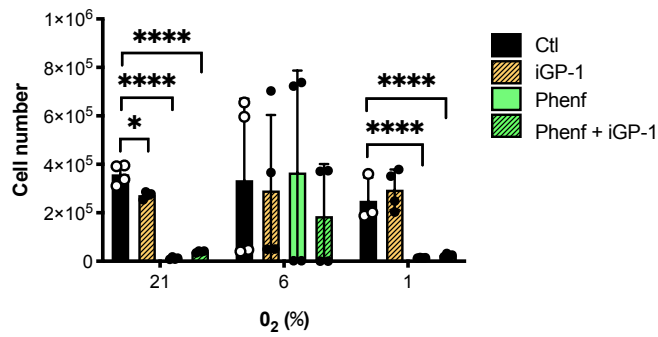**D****Group 3: D-458**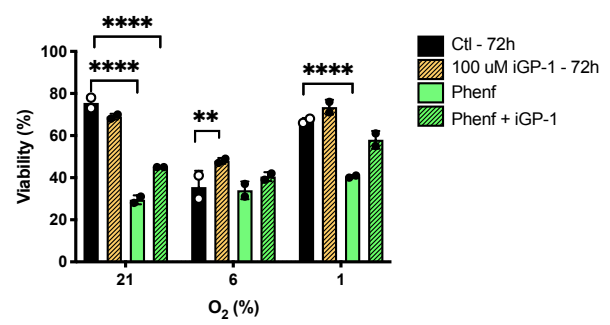

Supplement: Supplementary file 8 — Suppl. Figure 5 [file 41419_2023_6275_MOESM8_ESM.pdf]

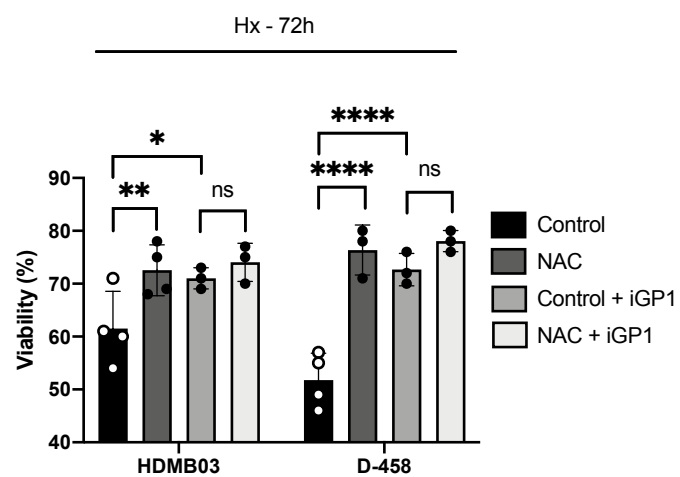

Supplement: Supplementary file 9 — Suppl. Figure 6 [file 41419_2023_6275_MOESM9_ESM.pdf]
